# Lymphocytic Esophagitis Mimicking Allergic Esophagitis: A Case Report

**DOI:** 10.7759/cureus.100033

**Published:** 2025-12-24

**Authors:** Rahul Reddy Nallapareddy, Hassan Muntazir, Jasbir Makker

**Affiliations:** 1 Internal Medicine, BronxCare Health System, New York City, USA; 2 College of Medicine, Boston University, Boston, USA; 3 Gastroenterology, BronxCare Health System, New York City, USA

**Keywords:** gastro-esophageal reflux disease, gerd, le, lymphocytic esophagitis, ppi, proton pump inhibitor

## Abstract

Lymphocytic esophagitis (LE) is a rare inflammatory condition of the esophagus, characterized by intraepithelial lymphocytic infiltration of the esophageal mucosa. Despite its increasing recognition recently, standardized guidelines for diagnosis and management are still lacking. LE is frequently associated with conditions that alter esophageal pH, including gastroesophageal reflux disease (GERD), Crohn's disease, and diabetes mellitus. This report describes a 55-year-old male who presented with dysphagia and intermittent heartburn. Esophagogastroduodenoscopy (EGD) revealed LE along with incidental *Helicobacter pylori *gastritis.

Management typically focuses on addressing underlying conditions, with proton pump inhibitors (PPIs) and topical steroids being the most common therapeutic approaches. The discussion highlights the prevalence, diagnostic challenges, and treatment strategies for LE.

## Introduction

Lymphocytic esophagitis (LE) is a relatively recently recognized inflammatory disorder of the esophagus characterized by an increased number of lymphocytes within the squamous epithelium. First described about two decades ago, LE has emerged as a distinct endoscopic and histopathological phenotype of chronic esophagitis, although its pathogenesis and clinical significance are still being elucidated. Unlike more well-established esophageal inflammatory conditions such as eosinophilic esophagitis, which is driven by eosinophil-mediated allergic inflammation, LE is defined by lymphocyte-predominant injury without significant granulocytic infiltration.

The esophageal mucosa is a multilayered squamous cell tissue whose surface is protected by the alkaline pH of the secretions of salivary glands and the esophagus. Hence, LE is mostly associated with conditions that lead to increased acid exposure in the esophagus, like gastroesophageal reflux disease (GERD), gastric ulcer, and hiatal hernia. Other associated conditions of LE are carcinoma of the esophagus, Crohn's disease, diabetes mellitus, cirrhosis, and hypertension.

Histologically, a mucosal biopsy from a patient with LE is characterized by intraepithelial lymphocytes (IELs) predominantly around peri-papillary fields. LE is currently being diagnosed by the criteria introduced by Rubio et al. However, there is still ambiguity around the diagnostic criteria and management of the condition [1,2].

## Case presentation

**Figure 1:**
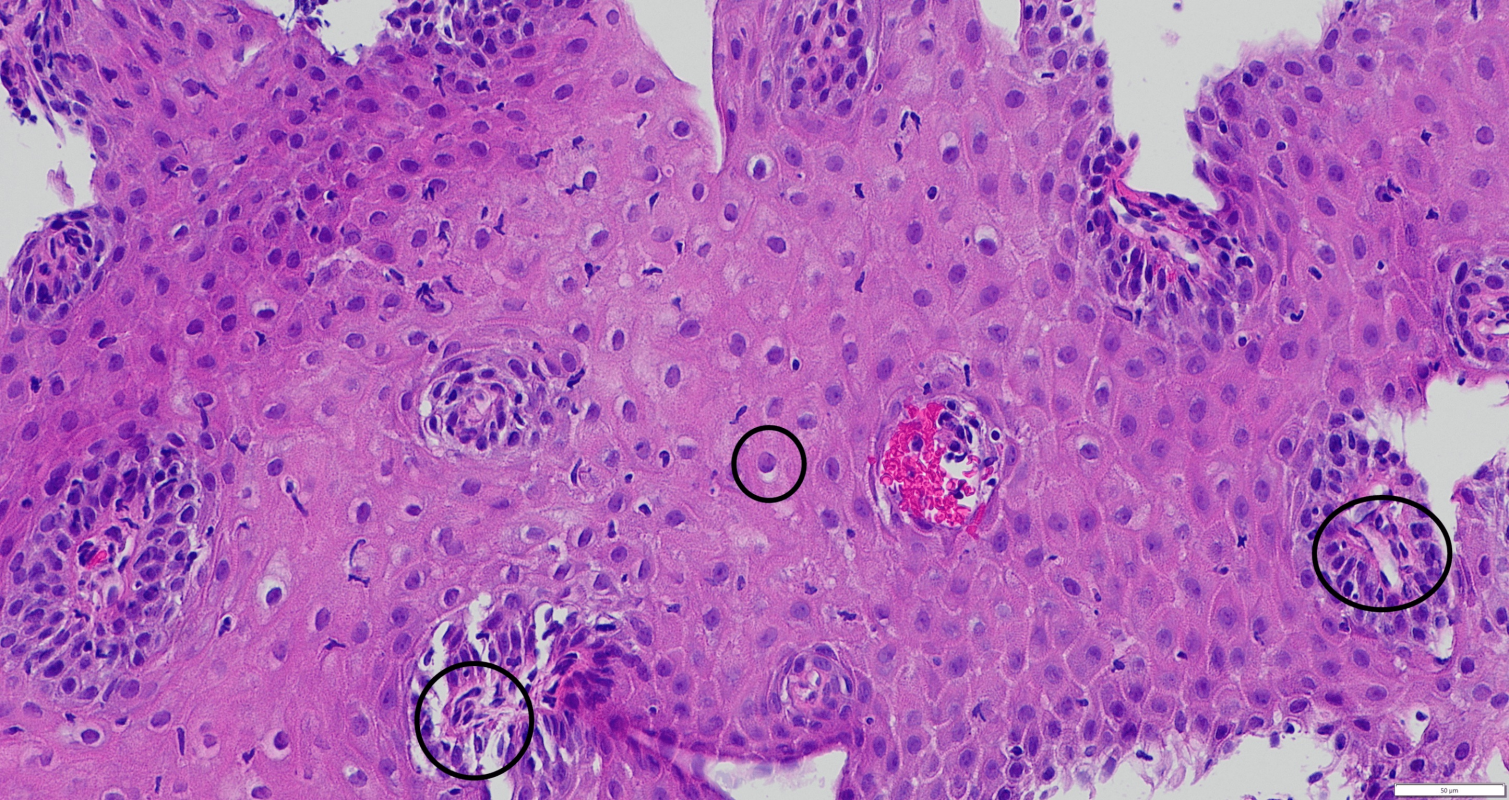
Histopathological examination of a biopsy specimen from the upper third of the esophagus at 10× magnification. The central circle highlights peri-papillary lymphocytic infiltration, the right circle indicates intracellular edema, and the left circle demonstrates intraepithelial lymphocytes.

**Figure 2:**
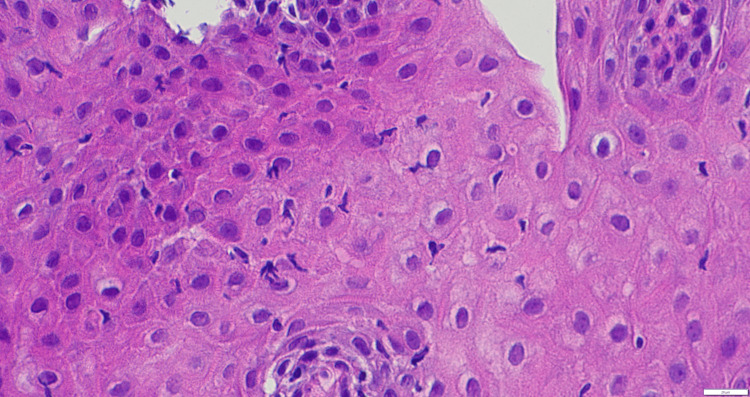
Intra-mucosal lymphocytes (up to 23 per high-power field) with no granulocytes or eosinophils, along with intracellular edema.

A 55-year-old man with a medical history of hypertension, diabetes mellitus, and hyperlipidemia had been initially referred for evaluation of dysphagia. For the past four months, he had experienced a sensation of something being lodged in his throat after eating, which subsided after a few hours. He also reported intermittent heartburn and acid reflux. Given the presence of GERD symptoms, the patient was recommended to initiate treatment with a proton pump inhibitor (PPI) and planned for esophagogastroduodenoscopy (EGD), which showed a normal esophagus and diffuse moderately erythematous gastric mucosa. Biopsies from the upper and lower esophagus showed LE with intra-mucosal lymphocytes (up to 23 per high power field) with no granulocytes or eosinophils and intracellular edema. Erythematous gastric mucosa biopsy revealed *Helicobacter pylori*. The patient was given a two-week bismuth quadruple antibiotic treatment for *H. pylori* and an eight-week course of PPI, resulting in complete resolution of his symptoms as well as eradication of *H. pylori*. Another follow-up EGD after completing eight weeks of PPI treatment showed remarkable improvement with no lymphocytes seen on histologic examination of random esophageal mucosal biopsies (Figures 1-2).

## Discussion

Disease prevalence

Studies have shown that the prevalence of LE varies depending on the presenting complaint and associated condition. A clinicopathologic study of patients with esophageal biopsies reported that 0.1% of patients with esophageal biopsies were diagnosed with LE, with dysphagia being the presenting symptom, and a higher prevalence was noted among women [3]. However, a retrospective study involving 238 patients with esophageal food bolus impaction reported a higher prevalence in men, with the average age of onset occurring in the fifth decade of life [4].

In the pediatric age group, the prevalence of LE has been reported at 5.7% [5]. A blind histologic review of 580 biopsies from 545 unique patients with LE noted a higher prevalence among children with Crohn’s disease than in adults with the same condition [5]. Crohn’s disease is one of the most common conditions associated with LE, especially in the pediatric age group. A study by Purdy et al. showed that 38% of patients with LE also had Crohn’s disease [6]. However, such an association was not observed in adults with Crohn’s disease [5,7].

Pathophysiology

The esophagus is composed of multiple layers of squamous cells that are protected by alkaline secretions of saliva and the esophagus itself. Conditions that lower the pH of the esophageal lumen, most notably acid reflux, are frequently associated with LE. Acid reflux introduces gastric contents into the esophageal lumen, serving as a persistent irritant. Chronic exposure of the esophagus to gastric acid leads to basal cell hyperplasia and abnormally tall papillae, as the basal layer of the squamous epithelium proliferates in response. Granulocytes such as neutrophils and eosinophils may infiltrate the epithelium as well [8].

In earlier studies on esophagitis, intraepithelial infiltration of lymphocytes was observed in the biopsies of esophageal biopsies, but was not initially considered a diagnostic criterion for esophagitis [9-11]. For instance, Butt and co-authors in their study on allergic esophagitis found that infants with cow-milk induced esophagitis exhibited predominant eosinophil eotaxin expression in the basal and papillary epithelium, with a focal lymphocyte activation [10]. Mangano et al. reported that esophageal biopsies from patients with reflux often had intraepithelial cells with irregular nuclear contours [12]. Similarly, Rubio and Hubbard, while reviewing the baboon gastric tissue for Menetrier’s disease and varioliform lymphocytic gastritis, noted the presence of lymphocytes with both round and irregular nuclear contours in the esophageal epithelium but no granulocyte infiltration [13].

Since these early observations, several case reports and series have documented intraepithelial lymphocytic infiltration in human esophageal biopsies associated with a variety of symptoms and underlying conditions [1]. In 2006, Rubio et al. published a landmark series describing 20 cases of a unique form of chronic esophagitis characterized by a high number of IELs, particularly in peripapillary fields. Notably, the lymphocyte density was significantly lower in the interpapillary regions, a distribution pattern that was in contrast with other esophagitis types such as GERD, *Candida*-related esophagitis, and radiation esophagitis [8,14].

Diagnosis and histopathology

The diagnosis of LE is most commonly made using a three-zone biopsy protocol, which includes biopsies collected from the proximal, middle, and distal esophagus. Various studies have used different cutoffs, ranging from >10 IELs to >100 IELs, with the most widely accepted number being >20 IEL [15]. These IELs are predominantly located in the peri-papillary fields (Figure 1). Immunohistochemical staining has demonstrated that most of these lymphocytes are CD3+ T lymphocytes, many of which also express CD8. Interestingly, studies investigating IELs in patients with esophageal motility disorders have reported a reverse CD4:CD8 ratio, with a predominance of CD4+ T cells [16].

Some studies have also reported spongiosis as a diagnostic feature of LE (Figure 2). Although many studies rely on predominance of lymphocytes and the absence of granulocytes for diagnosis, only a few specify an actual cutoff of <14 granulocytes per high-power field (hpf) as a diagnostic criterion for LE [15].

Endoscopic features

Endoscopic features in LE are variable. A systematic review involving 387 patients reported that 31% had a normal appearing esophagus on EGD [14]. In others, findings included erosive esophagitis, multiple esophageal rings, linear furrows, and strictures [11].

Treatment

Until the date of writing this case report, there are no formal guidelines available for the treatment of LE [17]. Management is generally directed towards the presenting symptom and the underlying or associated conditions. Treatment approaches are similar to those used for eosinophilic esophagitis and primarily involve two therapies: PPIs and topical corticosteroids.

The precise mechanism of action of PPIs remains unclear. It may involve both antisecretory effects via blockage of the hydrogen-potassium adenosine triphosphatase (H^+^/K^+^-ATPase) gastric enzyme, as well as anti-inflammatory effects. PPIs are not effective in all patients, as evidenced by studies reporting their benefit only in a quarter of patients, particularly those with GERD or motility disorders [1,7]. In patients with underlying autoimmune disorders, PPIs were generally ineffective and further delayed treatment response [18]. Topical steroids, such as swallowed fluticasone [19] or budesonide [18], have demonstrated clinical benefit and are increasingly being used to manage LE, especially when PPIs fail.

Once LE progresses to esophageal strictures, topical steroids alone are insufficient, and endoscopic dilatation becomes necessary. A case series involving 62 cases reported that 11% of LE patients developed esophageal strictures, and combination therapy with endoscopic dilation and medical therapy was beneficial in such cases [20].

Other therapies and considerations

In cases where LE is associated with underlying autoimmune, motility, and inflammatory disorders, addressing the primary condition is essential to achieving symptom control [1]. A case report described successful treatment of steroid- and PPI-refractory LE with vedolizumab, a monoclonal antibody targeting α4β7 integrin, which inhibits lymphocyte migration, suggesting potential for biologic therapy in select, treatment-resistant patients [21].

## Conclusions

LE is a rare esophageal disorder characterized by increased IELs and minimal granulocytic infiltration, predominantly CD3+ T cells, and in a peri-papillary distribution. The prevalence and associated conditions vary by age and comorbidities. LE is notably linked to pediatric Crohn’s disease, as well as conditions that lower esophageal pH, such as GERD. Diagnosis relies on histological examination of esophageal biopsy, since endoscopic findings are often nonspecific or even normal, much like eosinophilic esophagitis, which is the most common differential diagnosis. Currently, there are no standardized guidelines for LE diagnosis and management. The most common treatment approaches include PPIs and topical steroids, with endoscopic dilation reserved for patients with strictures. Further research is needed to better define its pathogenesis, establish diagnostic criteria, and optimize management strategies.
